# The Effect of Physical Resistance Training on Baroreflex Sensitivity of Hypertensive Rats

**DOI:** 10.5935/abc.20170065

**Published:** 2017-06

**Authors:** Moisés Felipe Pereira Gomes, Mariana Eiras Borges, Vitor de Almeida Rossi, Elizabeth de Orleans C. de Moura, Alessandra Medeiros

**Affiliations:** Universidade Federal de São Paulo (UNIFESP), São Paulo, SP - Brazil

**Keywords:** Hypertension, Exercise, Heart Rate, Baroreflex, Muscle Hypertrophy

## Abstract

**Background:**

Baroreceptors act as regulators of blood pressure (BP); however, its sensitivity is impaired in hypertensive patients. Among the recommendations for BP reduction, exercise training has become an important adjuvant therapy in this population. However, there are many doubts about the effects of resistance exercise training in this population.

**Objective:**

To evaluate the effect of resistance exercise training on BP and baroreceptor sensitivity in spontaneously hypertensive rats (SHR).

**Method:**

Rats SHR (n = 16) and Wistar (n = 16) at 8 weeks of age, at the beginning of the experiment, were randomly divided into 4 groups: sedentary control (CS, n = 8); trained control (CT, n = 8); sedentary SHR (HS, n = 8) and trained SHR (HT, n = 8). Resistance exercise training was performed in a stairmaster-type equipment (1.1 × 0.18 m, 2 cm between the steps, 80° incline) with weights attached to their tails, (5 days/week, 8 weeks). Baroreceptor reflex control of heart rate (HR) was tested by loading/unloading of baroreceptors with phenylephrine and sodium nitroprusside.

**Results:**

Resistance exercise training increased the soleus muscle mass in SHR when compared to HS (HS 0.027 ± 0.002 g/mm and HT 0.056 ± 0.003 g/mm). Resistance exercise training did not alter BP. On the other hand, in relation to baroreflex sensitivity, bradycardic response was improved in the TH group when compared to HS (HS -1.3 ± 0.1 bpm/mmHg and HT -2.6 ± 0.2 bpm/mmHg) although tachycardia response was not altered by resistance exercise (CS -3.3 ± 0.2 bpm/mmHg, CT -3.3 ± 0.1 bpm/mmHg, HS -1.47 ± 0.06 bpm/mmHg and HT -1.6 ± 0.1 bpm/mmHg).

**Conclusion:**

Resistance exercise training was able to promote improvements on baroreflex sensitivity of SHR rats, through the improvement of bradycardic response, despite not having reduced BP.

## Introduction

According to the World Health Organization, hypertension is a major risk factor related to death and disability worldwide, affecting billions of people and killing about 9.4 million individuals every year.^1^ In Brazil, about 31 million people are hypertensive, disease responsible for 1,683 in-hospital deaths.^2^

Hypertension occurs when the body loses the ability to maintain homeostasis of blood pressure (BP). The human body has many different mechanisms for BP control, among them: central nervous system ischemic response, renin-angiotensin-aldosterone system and the baroreflex system.^3,4^ The baroreflex system consists in receptors located in the carotid arteries and aorta, which are sensitive to BP changes.^5^ When there is an elevation in BP, baroreceptors send a signal to the nucleus of the solitary tract, which, in turn, excites the caudal ventrolateral medulla, inhibiting the premotor neurons of the rostral ventrolateral medulla, thus decreasing the cardiac contractility and consequently the BP. However, when there is a decrease in BP, baroreceptors increase sympathetic activity by decreasing the transmission of inhibitory signals to the pressure-regulating center. But, when BP is continuously high, an adaptive response of these receptors occurs, which shifts the normal BP threshold upward, making this regulatory system ineffective to deal with abnormal pressures.^6,7^

In order to reduce BP levels and health problems, the main guidelines advocate lifestyle changes, through nutritional education and physical activity as recommendations to everyone, while drug therapy should be used only by patients diagnosed with hypertension or with borderline hypertension with high global cardiovascular risk.^8^

Several studies have shown that aerobic exercise training of mild or moderate intensity is effective in reducing BP by improving baroreflex control of HR significantly in hypertensive rats, as well as controlling risk factors associated with hypertension.^9-11^ Although there is no consensus in the literature on the effects of resistance training on BP,^12-14^ practicing this type of training can be beneficial to hypertensive patients, especially elderly people, since muscle strength decreases with age, thus decreasing the quality of life.^15^ Therefore, the aim of this study was to evaluate the effect of resistance exercise training on BP and the sensitivity of baroreceptor in spontaneously hypertensive rats (SHR).

## Methods

### Reagents

Epinephrine (Sigma-Aldrich Co., USA), sodium nitroprusside (Sigma-Aldrich Co., USA) and potassium chloride (Synth).

### Animals

SHR (n = 16) and Wistar rats (n = 16) were obtained from the CEDEME (Center for the Development of Experimental Models for Biology and Medicine) at the University UNIFESP. All rats were male and 8 weeks of age at the beginning of the experiment. Cages held four animals each, and the animals were fed with a standard diet for laboratory rodents (Nuvilab) and water *ad libitum*. Room temperature was kept between 22-23°C and a light/dark cycle of 12:12 hours was adopted, with the light period beginning at 8:00 a.m. All experiments were carried out in accordance with National Research Council's Guidelines for the Care and Use of Laboratory Animals and were conducted after approval by the Ethics and Research Committee of the UNIFESP (CEP #0233/12). The animals were randomly divided into four groups, as follows: sedentary control (CS, n = 8); trained control (CT, n = 8); sedentary SHR (HS, n = 8) and trained SHR (HT, n = 8).

### Murinometrics and evaluated vital signs

The body mass in all groups was evaluated in semi-analytical balance (Gehaka), in the last day of experimental protocol, before the animals were anesthetized for euthanasia. The BP was evaluated by tail plethysmography (1day/week, during 8 weeks) using a specific system for rats (Visitech Systems: BP-2000 - Series II - Blood Pressure Analysis System) on days that the rats were not subjected to training session.

### Training protocol

After adaptation, all animals were habituated to the act of climbing steps for 5 consecutive days before the maximal load test. The test consisted of an initial load of 75% of the body mass, which was attached to the base of the tail. The load was progressively increased by 50 g increments in subsequent climbs.^16^ The resistance exercise training was then performed using the normalized value of the individual maximal load (load of the last complete climb/body weight) for each rat, and was adjusted in the fourth week according to the new test maximal load. Resistance exercise was performed 5 days/week, during 8 weeks at moderate intensity (40-60% of maximal load). The rats performed 15 climbs per session with a 1-min interval between climbs.^16^

### Baroreflex sensitivity

48 hours after the last exercise session, the animals were anesthetized with xylazine (20 mg/kg, ip) and ketamine (40 mg/kg, ip) and catheters made of polyethylene tubing PE-10 and PE-50 (Clay Adams, Parsipanny, NJ, USA) were introduced into carotid artery and vein. Mean arterial pressure (MAP) and heart rate (HR) were registered online, 48 hours after the last training session, through an analog-digital plate PowerLab (ADInstruments, Australia). The baroreflex control of HR was evaluated by bradycardia responses (vagal component) compared to a pressor and tachycardia stimulation (sympathetic component) after a depressant stimulus. This was accomplished by the administration of bolus doses of epinephrine (3, 5 and 10 µg - ev) and depressor dose of sodium nitroprusside (5, 15 and 20 µg - ev), respectively, with 10-minute interval between doses.

Cardiac baroreflex gain was determined by the ratio of the ΔHR/ΔMAP induced by vasoactive drugs, and thus expressed as heart beats per millimeter of mercury (bpm/mmHg).

### Euthanasia

The animals were deeply anesthetized with urethane (1.7 g/kg - ev) followed by administration of 5% KCl (ev). The soleus and extensor digitorum longus (EDL) were removed for weighing the masses and had their values corrected by tibial length.

### Statistical analysis

The statistical analysis was performed in GraphPad Prism 5.0. The distribution of the data obtained in this study was verified by Shapiro-Wilk test. The data showed Gaussian distribution and were presented as mean ± standard error of the mean and compared using analysis of variance. MAP, body mass, muscle mass and HR were analyzed with analysis of variance (ANOVA), followed by post-hoc Tukey's tests. Systolic blood pressure (SBP), diastolic blood pressure (DBP) and the maximum load tests were analyzed by two-way ANOVA followed by post-hoc Bonferroni's tests. In all analyses, statistical significance was established when p < 0.05.

## Results

### Murinometrics and evaluated vital signs

**Figure 1:**
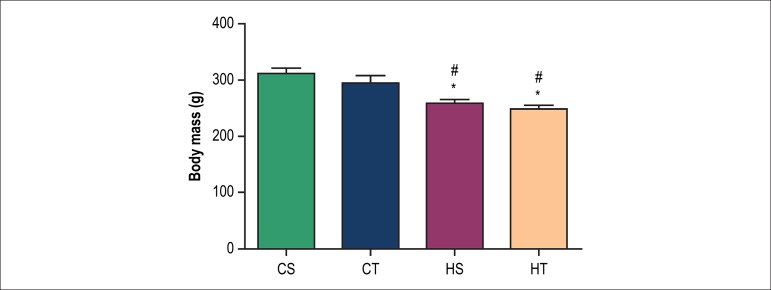
Body mass in grams (g) in sedentary control (CS), trained control (CT), sedentary SHR (HS) and trained SHR (HT) after 8 weeks of either sedentary or resistance exercise training protocol. * p < 0.05 vs. CS; # p < 0.05 vs. CT.

At the end of the experimental protocol, hypertensive animals (HS and HT) showed a decrease in body weight compared to the control groups (CS and CT). However, there was no significant change between HS and HT (Figure 1).

**Figure 2:**
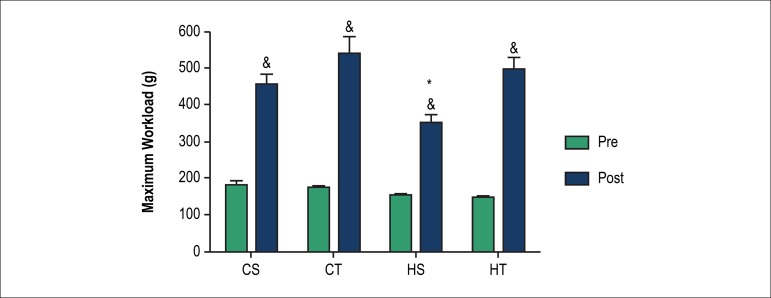
Maximum load test in grams (g) in sedentary control (CS), trained control (CT), sedentary SHR (HS) and trained SHR (HT) pre and post 8 weeks of either sedentary or resistance exercise training protocol. & p < 0.05 vs. same group at pre moment; * p < 0.05 vs. all groups at post moment.

At the end of the eighth week of exercise training protocol, it was possible observe the significant increase in the maximum strength in all groups. In addition, we could see that tolerance to weight at the end of the experimental protocol was lower in the HS group in comparison to other groups (Figure 2).

**Figure 3:**
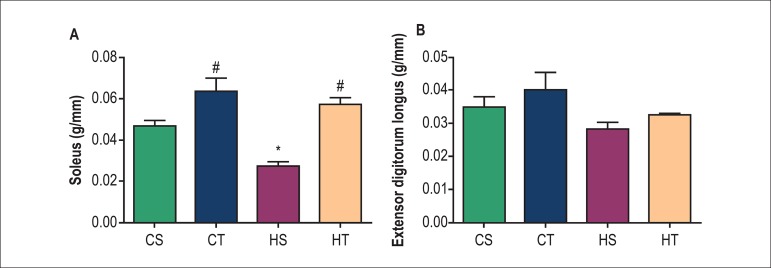
Muscle mass in grams corrected by tibia length (g/mm) in sedentary control (CS), trained control (CT), sedentary SHR (HS) and trained SHR (HT) after 8 weeks of either sedentary or resistance exercise training protocol. A) Soleus mass; B) Extensor digitorum longus mass. * p < 0.05 vs. CS; # p < 0.05 vs HS.

The mass of the soleus muscle in the HS group (0.027 ± 0.002 g/mm) was lower compared to the CS group (0.046 ± 0.005 g/mm, Figure 3A). Although the exercise has promoted increased muscle mass in the trained groups, only the HT group showed significant increase in relation to its control (0.056 ± 0.003 g/mm HT). Regarding the EDL muscle, there were no significant differences between groups (Figure 3B).

**Figure 4:**
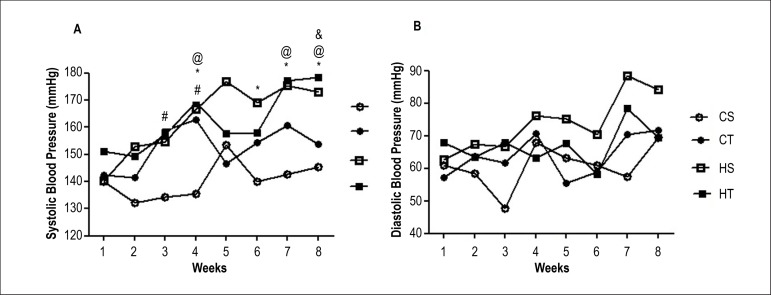
Blood Pressure measurements in sedentary control (CS), trained control (CT), sedentary SHR (HS) and trained SHR (HT) during or after 8 weeks of either sedentary or resistance exercise training protocol. A) Systolic blood pressure in mmHg evaluated by tail plethysmography; B) Diastolic blood pressure in mmHg evaluated by tail plethysmography. (*) There was significant difference between the groups HS vs. CS (p < 0.05); (#) There was significant difference between the groups CT vs. CS (p < 0.05); (@) There was significant difference between the groups HT vs. CS (p < 0.05); (&) There was significant difference between the groups CT vs. HT (p < 0.05). Significance based on two-way ANOVA with Bonferroni's post-hoc test.

Figure 4 shows the indirect measurements SBP and DBP over the eight weeks of the experimental protocol, and demonstrate the MAP and HR of the animals directly at the end of the protocol. Figure 4A demonstrates that the hypertensive groups (HS and HT) showed a significant increase in SBP from the fourth week compared to the CS group. Although the blood pressure of the CT group was higher compared to the CS in the third and fourth week, pressure levels showed no significant difference from the fifth week onwards. It was observed that the HT group showed significant reductions in SBP for two weeks (5th and 6th week), however, the decrease in SBP was not sustained.

**Figure 5:**
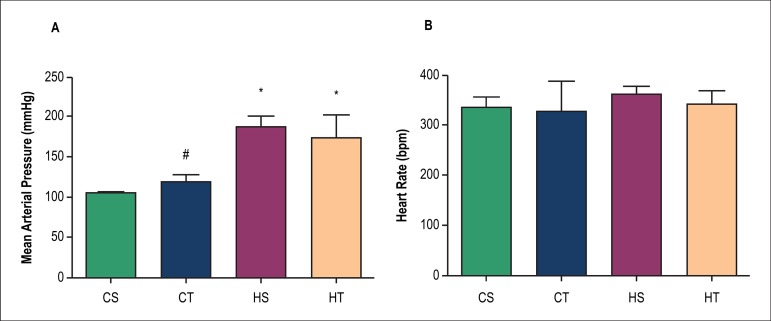
Mean arterial pressure and heart rate evaluated directly after experimental protocol. A) Mean arterial pressure (*) p < 0.05 vs. CS. (#) p < 0.05 vs. CT. and B) Heart rate. Significance based on one-way ANOVA with Tukey's post-hoc test. There was no significant difference.

There were no significant differences in DBP (Figure 4B). Figure 5A shows that MAP of HS group (188 ± 14 mmHg) and HT group (174 ± 29 mmHg), assessed directly, showed significantly higher values in comparison to the CS group (106 ± 3 mmHg). Resistance exercise training had no effect on MAP in groups CT or HT. No significant differences were found in the HR evaluated directly in animals (CT = 336 ± 20 bpm, HS = 362 ± 17 bpm, CT = 328 ± 60 bpm and HT = 342 ± 27 bpm), (Figure 5B).

### Baroreflex sensitivity

**Figure 6:**
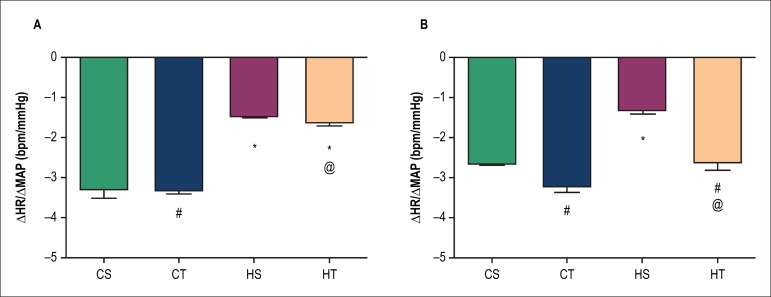
Baroreflex sensitivity (difference between ΔHR and ΔMAP) in sedentary control (CS), trained control (CT), sedentary SHR (HS) and trained SHR (HT) after 8 weeks of either sedentary or resistance exercise training protocol. A) Tachycardic sensitivity; B) Bradycardic sensitivity. * p < 0.05 vs. CS; # p < 0.05 vs. respective control group; @ p < 0.05 vs. CT. HR: heart rate; MAP: mean arterial pressure.

We found that exercise was not effective in promoting improved tachycardia sensitivity in hypertensive groups (CS -3.3 ± 0.2 bpm/mmHg, CT -3.3 ± 0.1 bpm/mm Hg, HS -1.47 ± 0.06 bpm/mmHg, HT -1.6 ± 0.1 bpm/mmHg) (Figure 6A). In relation to the bradycardic response, we observed a decrease in the HS group (-1.3 ± 0.1 bpm/mmHg) compared to the CS group (-2.67 ± 0.06 bpm/mmHg). Moreover, mean values of ΔHR/ΔMAP and bradycardic sensitivity were also higher in the HT group (-2.6 ± 0.2 bpm/mmHg) in relation to the HS group (Figure 6B).

## Discussion

It was found that hypertensive animals showed a decrease in body mass compared to control groups, and that resistance exercise training did not promote changes. The HS group also presented a reduced mass of the soleus muscle when compared to the CS group. Although it had no effect on body mass, resistance exercise training promoted an increase in soleus muscle mass in the HT group in comparison to the HS group. Therefore, we can infer that the lower body mass observed in HT rats was due to the likely reduction of adipose tissue, since resistance training promotes increased expression of genes related to lipid catabolism.^17,18^

The HS group showed less strength compared to the other groups in maximum load test; however, the resistance exercise training promoted increase in muscle strength after the exercise training period in both groups. Recent studies in humans have shown that there is a strong correlation between decreased handgrip performance with hypertension.^19-21^ Previous studies have related functional alteration of skeletal muscle with decreased nitric oxide bioavailability caused by increased reactive oxygen species (ROS), endothelin receptor type A and increased activation of protein catabolism due to increased angiotensin- II (ANG II).^22-25^ Such changes may explain the decrease in strength that was observed in the HS group. Concerning the increase in strength observed in all groups, when we compare post-experimental to pre-experimental data, animal growth can be cited as a factor responsible for this increase, as well as the adaptation of the animals to the test.

In fact, resistance exercise training is able to promote increased muscle mass, especially in the soleus.^26^ However, there were no significant changes in the EDL, as observed in other studies.^27,28^ According to Neves et al.,^28^ the type of training can justify these results, since the climbing training exercises promote little action in the EDL muscle and greater action in the soleus, because of the greater need of force employed by the rat to perform plantar flexion while up the stairs. A fact that contributes to this hypothesis is that training with electrical stimulation for muscle contraction promotes significant increase in mass of the EDL, while the soleus presents atrophy with this type of stimulus.^29^

Regarding hemodynamic parameters, it was found that the SHR animals developed spontaneous hypertension, with significant increase in SBP from the fourth week of the experimental protocol and thirteenth week of life, which was expected for the model as reported by other authors.^30-32^ Elevation of SBP observed in CT group at 3rd and 4th week of training is possibly related to the stress of the load that was increased in the half time of the training protocol and due to the beginning of the reproductive phase of the animals, between the 10^th^ and 12^th^ week of life, since testosterone increases the ANG II sensitivity.^33,34^

At the end of the experimental protocol, resistance exercise training did not promote alterations in MAP measured directly. Previous studies also found no significant effects of resistance training on BP.^35-37^

About the baroreflex sensitivity, it was found that the HS group showed a reduction in both bradycardic and tachycardia response, which was expected, as was noted earlier in experimental models^38,39^ and in humans.^40^ Resistance exercise training was able to promote significant improvement only in the bradycardic response. When analyzing the effect of resistance exercise training in rats with metabolic syndrome induced by hypercaloric diet, Valenti et al.^41^ obtained similar results to ours, demonstrating that this type of exercise is ineffective in improving the tachycardic response, regardless of the experimental model. Thus, the resistance exercise training seems to work mainly with the improvement of the sensitivity of the carotid baroreceptors, since the bradycardic response demonstrates strong correlation with the integrity of carotid sinus.^38-40^ Furthermore, increased bradycardic response collaborates with decreasing sympathetic activity in the heart, leading to a reduction in HR at rest, decreasing cardiac output and finally decreasing BP.^42^

## Conclusion

With the data obtained in this study, we can conclude that resistance exercise training, despite not promoting a significant decrease in BP in SHR, improves bradycardic response. However, more studies are needed to understand the mechanisms that lead to this improvement.
